# Relationships among medication adherence, lifestyle modification, and health-related quality of life in patients with acute myocardial infarction: a cross-sectional study

**DOI:** 10.1186/s12955-018-0921-z

**Published:** 2018-05-22

**Authors:** Yu-Mi Lee, Rock Bum Kim, Hey Jean Lee, Keonyeop Kim, Min-Ho Shin, Hyeung-Keun Park, Soon-Ki Ahn, So Young Kim, Young-Hoon Lee, Byoung-Gwon Kim, Heeyoung Lee, Won Kyung Lee, Kun Sei Lee, Mi-Ji Kim, Ki-Soo Park

**Affiliations:** 1 Department of Preventive Medicine, School of Medicine, Kyungpook National University, Daegu, South Korea; 2 Gyeongnam Regional Cardiocerebrovascular Disease Center, Gyeongsang National University Hospital, Jinju, South Korea; 3 Gangwon Regional Cardiocerebrovascular Disease Center, Kangwon National University Hospital, Chuncheon, South Korea; 4 Department of Preventive Medicine, Chonnam National University Medical School, Gwangju, Republic of Korea; 5 Jeju Regional Cardiocerebrovascular Disease Center, Jeju National University Hospital, Jeju, South Korea; 6 Daejeon-Chungnam Regional Cardiocerebrovascular Center, Chungnam National University Hospital, Daejeon, South Korea; 7 Chungbuk Regional Cardiocerebrovascular Disease Center, Chungbuk National University Hospital, Cheongju, South Korea; 8 Department of Preventive Medicine &Institute of Wonkwang Medical Science, Wonkwang University School of Medicine, Iksan, South Korea; 9 Busan-Ulsan Regional Cardiocerebrovascular Disease Center, Dong-A University Hospital, Busan, South Korea; 10 Center for preventive medicine and public health, Seoul National University Bundang Hospital, Seongnam, South Korea; 11 Department of Social and Preventive Medicine, Inha University School of Medicine, Incheon, South Korea; 12 Department of Preventive Medicine, School of Medicine, Konkuk University, Seoul, South Korea; 13 Department of Preventive Medicine, College of Medicine and Institute of Health Science, Gyeongsang National University, 15 Jinju-daero, 816 Beon-gil, Jinju, 52727 South Korea

**Keywords:** Myocardial infarction, Health behavior, Lifestyle modification, Medication adherence, Secondary prevention, Health-related quality of life, Healthy adherer effect

## Abstract

**Background:**

The healthy adherer effect is a phenomenon in which patients who adhere to medical therapies tend to pursue health-seeking behaviors. Although the healthy adherer effect is supposed to affect health outcomes in patients with coronary artery disease, evaluation of its presence and extent is not easy. This study aimed to assess the relationship between medication adherence and lifestyle modifications and health-related quality of life among post-acute myocardial infarction (AMI) patients.

**Methods:**

A cross-sectional study was conducted in 417 post-AMI patients who underwent percutaneous coronary intervention (PCI). Patients were recruited from 11 university hospitals from December 2015 to March 2016 in South Korea. Details regarding socio-demographic factors, six health behaviors (low-salt intake, low-fat diet and/or weight-loss diet, regular exercise, stress reduction in daily life, drinking in moderation, and smoking cessation), medication adherence using the Modified Morisky Scale (MMS), and HRQoL using the Coronary Revascularization Outcome Questionnaire (CROQ) were surveyed in a one-on-one interview.

**Results:**

In the univariate logistic analysis, sex (female), age (≥70 years), MMS score (≥5), and CROQ score were associated with adherence to lifestyle modification. In the multiple logistic analysis, a high MMS score (≥5) was associated with adherence to lifestyle modification after adjusting for sex, age, marital status, education, and family income (adjusted odds ratio [OR] = 11.7, 95% confidence interval [CI] = 1.5–91.3). After further adjusting for the CROQ score, the association between high MMS score and adherence to lifestyle modification was significant (adjusted OR = 11.5, 95% CI = 1.4–93.3).

**Conclusions:**

Adherence to medication was associated with adherence to lifestyle modification, suggesting the possible presence of the healthy adherer effect in post-AMI patients. After further adjusting for HRQoL, the association remained. To improve health outcome in post-AMI patients, early detection of patients with poor adherence to medication and lifestyle modification and motivational education programs to improve adherence are important. In addition, the healthy adherer effect should be considered in clinical research, in particular, in studies evaluating the effects of therapies on health outcomes.

**Electronic supplementary material:**

The online version of this article (10.1186/s12955-018-0921-z) contains supplementary material, which is available to authorized users.

## Background

In patients with acute myocardial infarction (AMI), comprehensive management of risk factors through lifestyle modification (e.g., smoking cessation, weight control, or physical activity) and evidence-based medical therapies are essential for the improvement of survival and for the prevention of recurrent cardiovascular events [1–4]. The phenomenon in which patients who adhere to therapies tend to pursue health-seeking behaviors is called the “healthy adherer effect”; it is a recognized problems in studies undertaken in order to evaluate the effects of medications on health outcomes [5]. This phenomenon has explained why cardiovascular outcomes from randomized clinical trials (RCTs) have had different outcomes from prior observational studies [5, 6]. For example, hormone replacement therapy was associated with reduced cardiovascular disease in a large observational study (the Nurses′ Health study); however, no cardiovascular benefit was seen in later RCTs [6].

Many other studies of the effect of medications on cardiovascular outcomes also have mentioned on this phenomenon. A study reported that a healthy adherer effect might partially lead to overestimation of beta-blockers’ true effects on the reduction of cardiovascular risks [7]. A retrospective cohort study on the effects of statin adherence concluded that the healthy adherer effect made some contributions to reduced occurrence of primary cardiovascular events among women [8]. In a 3-year longitudinal study of post-MI patients, however, adherence to guideline-recommended therapies was effective for lowering the rate of major adverse cardiovascular events (MACE) and for cost savings, even after adjusting for health-seeking behavior (flu vaccination) [9].

On the other hand, health-related quality of life (HRQoL), which is an important measure of health, is a strong predictor of mortality and hospital readmissions in patients with coronary artery disease [10–13]. Thus, the American Heart Association recommends that the inclusion of a patient’s HRQoL is an important measure of cardiovascular health among patients with cardiovascular disease [14]. HRQoL after percutaneous coronary intervention (PCI) is related to factors such as sex, age, anxiety and depressive symptoms, the number of diseased vessels, comorbidities, and pre-procedural angina frequency [15–18]. Medication adherence and lifestyle modification, two of the most important risk reduction recommendations among patients with cardiovascular disease, are related to HRQoL [19–21]. The physical health domain of HRQoL predicted adherence to the cardiac risk-reduction recommendations among AMI patients [20].

Numerous studies have addressed the issues regarding medication adherence and lifestyle modification after coronary artery disease [22–25]. However, the presence and extent of the healthy adherer effect, which is supposed to affect health outcomes, cannot be easily assessed in a study population [26]. In this study, we evaluated whether the relationship between medication adherence and lifestyle modification exists among post-AMI patients treated with percutaneous coronary intervention (PCI). To the best of our knowledge, no study is available to evaluate the association between medication adherence and lifestyle modification in relation to HRQoL. Thus, we also identified whether the relationship between medication adherence and lifestyle modification remains even after controlling for HRQoL.

## Methods

### Study design and population

A total of 417 participants were recruited from the cardiology outpatient clinic from the 11 university hospitals designated Regional Cardiocerebrovascular Centers (RCC) in South Korea; Kangwon, Daejeon-Chungnam, Daegu-Gyeongbuk, Gwangju-Jeonnam, Gyeongnam, Jeju, Busan-Ulsan, Jeonbuk, Chungbuk, Inchoen, and Gyeongi RCC. Clinical diagnosis and patient status were assessed by cardiologists at each hospital. Participants who were admitted to the hospital: 1) due to acute ST-segment elevation myocardial infarction (STEMI) and treated with PCI; and 2) within 12 to 15 months post-AMI, were included in this study. On the other hand, participants with medical conditions precluding reliable verbal communication were excluded. A one-on-one interview was conducted by trained nurses from the 11 hospitals from December 2015 to March 2016 using a structured questionnaire. Written informed consent was obtained from all participants prior to the start of the survey. The study was approved by the institutional review board of all the participating hospitals.

### Measurements

#### Socio-demographic factors and adherence to lifestyle modifications

The socio-demographic factors including age (years, continuous), sex (male/female, categorical), marital status (married/others, categorical), education (≤middle school/≥high school, categorical), family income (≤1/1–2/2–3/3–4/≥4 million won per month, categorical), and job status (employed/self-employed/unemployed, categorical) were surveyed. Based on the Medical Outcomes Study (MOS) Measures of Patient Adherence, our researchers selected six health behaviors (except for taking prescribed medication) that were appropriate for patients with AMI in Korea [27]. The participants were asked about the following six health behaviors: (1) low-salt diet, (2) low-fat diet and/or weight-loss diet, (3) regular exercise, (4) stress reduction in daily life, (5) drinking in moderation, and (6) smoking cessation. Patient’s responses were based on the following Likert scale: never, rarely, sometimes, very often, and always. Those who answered “very often” or “always” for each question were considered to have a high adherence to each health-related behavior.

#### Self-reported medication adherence

Adherence is defined as the extent to which the patient follows medical instructions [28]. The Morisky Scale is a self-reported measure of medication adherence [29]. The original four-item Morisky Scale was developed in order to help practitioners predict patient adherence to antihypertensive medications in the mid-1980s [30]. The validity of the English and Korean Morisky Scale has been discussed in detail elsewhere [29, 31]. To make up for shortcomings (e.g., cannot explain persistence of therapy) of the original Morisky Scale, the Case Management Society of America added two new questions to the Modified Morisky Scale (MMS) [32]. The six-item MMS measures two domains of adherence: knowledge and motivation. Because the MMS has been used in other study for Koreans [33] and can assess the two domains, we used the same MMS in this study that has been used in other studies. The score range is from 0 to 3 for the knowledge domain and from 0 to 3 for the motivation domain; thus the total score is from 0 to 6, and a high score indicates high adherence [32].

#### Health-related quality of life (HRQoL)

HRQoL of post-AMI patients was measured using the Coronary Revascularization Outcome Questionnaire (CROQ). The CROQ is a patient-based questionnaire that can specifically evaluate HRQoL and health outcomes before and after coronary revascularization, such as coronary artery bypass surgery (CABG) and percutaneous transluminal coronary angioplasty (PTCA) [34]. Compared to other generic or disease-specific measures of HRQoL for use among people with coronary heart disease, the CROQ is advantageous in that it can assess not only HRQoL but also adverse effects of coronary revascularization [34, 35]. Among four versions of the CROQ, the post-revascularization version (CROQ-PTCA_Post) was used in this study. The reliability and validity of the original and Korean version of the CROQ has discussed in detail elsewhere [34, 36]. The CROQ-PTCA_Post contains six subscales of items that measure symptoms (7 items), physical functioning (8 items), psychosocial functioning (14 items), cognitive functioning (3 items), satisfaction (6 items), and adverse effects (6 items). Each item is scored on a 3- to 6-point Likert scale; thus, the total score of the CROQ ranges from 0 to 100. A high score indicates a good outcome.

### Statistical analysis

The socio-demographic factors of participants were described based on the frequency (n) and proportion (%) for the categorical variables and mean with standard deviation for the continuous variables. The frequencies of higher adherence to lifestyle modification in relation to the socio-demographic factors were compared using the chi-square test. A multiple logistic regression analysis was used to examine the association between medication adherence and lifestyle modification after adjusting for covariates. In a multiple logistic regression analysis, the binary outcome variable was adherence to lifestyle modification (yes/no), defined as a subject who is adherent to five or more among the six lifestyle modification behaviors (i.e., low-salt diet, low-fat diet and/or weight-loss diet, regular exercise, stress reduction in daily life, drinking in moderation, and smoking cessation). Logistic model 1 included sex, age, marital status, education, family income, and total MMS score, while model 2 further included the CROQ score. All data analyses were performed using SAS version 9.4 (SAS Institute Inc., Cary, NC, USA). A *p*-value under 0.05 was considered statistically significant.

## Results

### General characteristics of study participants

The general characteristics of the study participants are presented in Table 1. Among the 417 participants, 83.5% were male, and the mean age was 62.4 ± 11.7 years. Additionally, 75.5% were married, 62.6% had received an education beyond high school graduation, 26.1% had a family income of less than 100 million won per month, and 37.4% were unemployed. The mean scores of the MMS were 2.5 ± 0.8 (motivation), 2.4 ± 0.6 (knowledge), and 4.9 ± 1.1 (total). The means of the CROQ scores were 75.4 ± 19.6 (psychosocial functioning), 84.3 ± 17.0 (cognitive functioning), 85.0 ± 15.2 (satisfaction), 86.6 ± 14.9 (symptoms), 90.2 ± 15.9 (physical functioning), and 91.7 ± 14.4 (adverse effect).

**Table 1:** The proportion of subjects with adherence to lifestyle modifications according based on the general characteristics (n=417)

				High adherence to lifestyle modification^a^ (%)		
Characteristics		n	(%)	yes	no	*p*-value
Sex	male	348	(83.5)	36.5	63.5	<0.001^*^
	female	69	(16.5)	58.0	42.0	
Age (years)	<50	61	(14.6)	23.0	77.0	0.008^*^
	50-59	108	(25.9)	37.0	63.0	
	60-69	126	(30.2)	42.9	57.1	
	70≤	122	(29.3)	48.4	51.6	
Marital status	married	315	(75.5)	42.2	57.8	0.111
	single/others^b^	102	(24.5)	33.3	66.7	
Education	≤ middle school	156	(37.4)	46.2	52.9	0.049^*^
	≥ high school	261	(62.6)	36.4	63.6	
Family income (10,000 won/month)	≤100	109	(26.1)	46.8	53.2	0.078
	101-200	71	(17.0)	47.9	52.1	
	201-300	95	(22.8)	34.7	65.3	
	≥301	142	(34.1)	34.5	65.5	
Job	employed	122	(29.3)	41.0	59.0	0.091
	self-employed	139	(33.3)	33.1	66.9	
	unemployed^c^	156	(37.4)	45.5	54.5	

^a^defined as a subject who are adherent to five or more among the six health behaviors including low-salt intake, low-fat diet and/or weight-loss diet, regular exercise, stress reduction in daily life, drinking in moderation, and smoking cessation

^b^others included separated, divorced, and widowed status

^c^unemployed included a housewife (n=9)

^*^p-value <0.05

### Medication adherence to and adherence to lifestyle modification

Adherence to lifestyle modification tended to be higher in females, participants of older age, and those who were married, had less education, had a low family income, and were unemployed (Table 1). MMS-total and MMS-knowledge were significantly associated with adherence to lifestyle modification (Table 2). Among the six lifestyle modifications, MMS-knowledge was associated with a low-fat diet and/or weight-loss diet (*p* < 0.01) and drinking in moderation (*p* < 0.01). On the other hand, MMS-motivation was significantly associated with a low-salt diet (*p* < 0.01), low-fat diet and/or weight-loss diet (*p* = 0.04), stress reduction in daily life (*p* < 0.01), drinking in moderation (*p* < 0.01), and smoking cessation (p < 0.01) (Table 2).

**Table 2:** The proportion of subjects with adherence to lifestyle modification according to the Modified Morisky Scale (MMS) score

		MMS score											
		Knowledge				Motivation				Total (knowledge + motivation)			
	State	0-1	2	3	*p*-value	0-1	2	3	*p*-value	0-2	3-4	5-6	*p*-value
n		17	207	193		49	91	277		18	70	329	
High adherence to lifestyle modification^b^ (%)	yes	17.6	44.0	37.8	0.072	20.4	33.0	45.8	0.001^*^	5.6	27.1	44.7	<0.001^*^
	no	82.4	56.0	62.2		79.6	67.0	54.2		94.4	72.9	55.3	
Individual lifestyle modification													
Low-salt diet^a^ (%)	yes	29.4	51.2	46.1	0.175	28.6	40.7	53.8	0.001^*^	16.7	37.1	52.0	0.002^*^
	no	70.6	48.8	53.9		71.4	59.3	46.2		83.3	62.9	48.0	
Low-fat diet and/or weight-loss diet^a^ (%)	yes	23.5	61.4	51.8	0.004^*^	40.8	51.6	59.2	0.042^*^	16.7	52.9	58.1	0.002^*^
	no	76.5	38.6	48.2		59.2	48.4	40.8		83.3	47.1	41.9	
Regular exercise^a^ (%)	yes	47.1	53.6	52.9	0.872	36.7	56.0	54.9	0.052	33.3	44.3	55.9	0.048^*^
	no	52.9	46.4	47.2		63.3	44.0	45.1		66.7	55.7	44.1	
Stress reduction in daily life^a^ (%)	yes	41.2	62.8	53.9	0.072	28.6	53.8	64.3	<0.001^*^	27.8	44.3	62.3	<0.001^*^
	no	58.8	37.2	46.1		71.4	46.2	35.7		72.2	55.7	37.7	
Drinking in moderation^a^ (%)	yes	35.3	76.8	77.7	<0.001^*^	46.9	73.6	81.2	<0.001^*^	27.8	65.7	80.2	<0.001^*^
	no	64.7	23.2	22.3		53.1	26.4	18.8		72.2	34.3	19.8	
Smoking cessation^a^ (%)	yes	52.9	76.3	78.2	0.063	63.1	73.6	81.2	<0.001^*^	38.9	68.6	79.9	<0.001^*^
	no	47.1	23.7	21.8		46.9	26.4	18.8		61.1	31.4	20.1	

^a^yes, Likert scale ≥4; no, Likert scale <4

^b^defined as a subject who are adherent to five or more among the six health behaviors including low-salt intake, low-fat diet and/or weight-loss diet, regular exercise, stress reduction in daily life, drinking in moderation, and smoking cessation

^*^p-value <0.05

In the unadjusted logistic analysis, adherence to lifestyle modification was significantly higher in females (crude odds ratio [OR] = 2.4, 95% confidence interval [CI] = 1.4–4.1), age ≥ 70 years (crude OR = 3.1, 95% CI = 1.6–6.3), subjects with high MMS-total (crude OR = 13.7, 95% CI = 1.8–104.3), and with a high CROQ score (5 subclasses except for physical functioning) (Table 3). In the multiple logistic analysis (model 1), a high MMS-total was associated with adherence to lifestyle modification (adjusted OR = 11.7, 95% CI = 1.5–91.3), after adjusting for sex, age, marital status, education, and family income. Even after further adjusting for six CROQ subscales (model 2), a high MMS-total was significantly associated with adherence to lifestyle modification (adjusted OR = 11.5, 95% CI = 1.4–93.3). Among the six CROQ subscales, improvement in CROQ-symptoms and CROQ-satisfaction was significantly associated with adherence to lifestyle modification in model 2.

**Table 3:** Odds ratios on the adherence to lifestyle modification using logistic regression analysis^a^ (n=417)

Characteristics		Unadjusted			Adjusted analysis model1			Adjusted analysis model2		
		OR	95% CI	*p*-value	OR	95% CI	*p*-value	OR	95% CI	*p*-value
Sex	male (ref)	1.00			1.00			1.00		
	female	2.40	1.42-4.06	0.001^*^	2.28	1.23-4.22	0.009^*^	2.29	1.19-4.42	0.013^*^
Age (years)	<50 (ref)	1.00			1.00			1.00		
	50-59	1.98	0.97-4.03	0.969	1.55	0.74-3.26	0.958	1.81	0.84-3.90	0.766
	60-69	2.52	1.26-5.04	0.162	1.79	0.82-3.88	0.411	1.97	0.88-4.39	0.457
	70≤	3.14	1.57-6.30	0.007^*^	2.00	0.87-4.60	0.213	2.37	0.99-5.66	0.150
Marital status	married	1.46	0.92-2.34	0.112	1.84	1.06-3.19	0.029^*^	1.63	0.99-5.66	0.093
	single/others ^b^ (ref)	1.00			1.00			1.00		
Education	≤ middle	1.50	1.00-2.24	0.050	1.12	0.66-1.89	0.670	1.63	0.92-2.89	0.401
	≥ high (ref)	1.00			1.00					
Family income (10,000won/month)	≤100 (ref)	1.00			1.00			1.00		
	101-200	1.05	0.57-1.90	0.146	1.14	0.58-2.21	0.265	1.04	0.73-2.19	0.380
	201-300	0.61	0.34-1.07	0.159	0.73	0.37-1.47	0.331	0.65	0.31-1.34	0.194
	≥301	0.60	0.36-1.00	0.096	0.76	0.39-1.51	0.430	0.78	0.38-1.60	0.660
MMS -total	0-2(ref)	1.00			1.00			1.00		
	3-4	6.33	0.79-50.87	0.358	5.01	0.61-41.36	0.521	4.69	0.55-40.14	0.592
	5-6	13.72	1.81-104.27	0.002^*^	11.69	1.50-91.29	0.003^*^	11.48	1.41-93.30	0.003^*^
CROQ-symptoms		1.03	1.02-1.05	<0.001^*^				1.03	1.01-1.04	0.011^*^
CROQ-physical functioning		1.01	0.99-1.02	0.309				1.00	0.98-1.02	0.922
CROQ-psychosocial functioning		1.01	1.00-1.03	0.009^*^				1.00	0.98-1.01	0.733
CROQ-cognitive functioning		1.01	1.00-1.03	0.046^*^				1.00	0.99-1.02	0.686
CROQ-satisfaction		1.02	1.01-1.04	<0.001^*^				1.02	1.00-1.04	0.018^*^
CROQ-adverse effects		1.02	1.00-1.04	0.017^*^				1.02	1.00-1.04	0.064

^a^binary outcome variable was higher adherence to lifestyle modifications, that was defined as a subject who are adherent to five or more among the six health behaviors including low-salt intake, low-fat diet and/or weight-loss diet, regular exercise, stress reduction in daily life, drinking in moderation, and smoking cessation; ^b^ others included separated, divorced, and widowed status

^*^p-value <0.05

In further analyses wherein the MMS was divided into two domains and put in the logistic model simultaneously, high MMS-motivation was significantly associated with adherence to lifestyle modification (adjusted OR = 3.0, 95% CI = 1.3–6.8) after adjusting for all covariates (Additional file 1: Table S1). In contrast with, with MMS-motivation, MMS-knowledge was not associated with adherence to lifestyle modification in an adjusted analysis.

## Discussion

Adherence to lifestyle modification was significantly associated with medication adherence in post-AMI patients after adjusting for sex, age, marital status, education level, and family income (model 1). This association remained even after further adjusting for health-related quality of life (model 2). This finding implies a possible presence of a healthy adherer effect in post-AMI patients, suggesting that patients with low medication adherence may have an unhealthy lifestyle. According to previous studies, a considerable number of post-AMI patients have low adherence to medication. For example, even as early as 6 weeks after AMI patients are treated with PCI, approximately 29% of them showed moderate or low adherence to prescribed cardiovascular medication [25]. In another study, over 25% of post-AMI patients did not fill their prescribed medicine after discharge, and the 1-year mortality rate was higher for patients who did not fill all of their discharge medications [37].

In addition to medication use, patients with AMI should improve their lifestyles, including smoking cessation, alcohol consumption, weight control, dietary control, and stress control, to improve their prognosis. However, most patients are experiencing difficulties in lifestyle modification compared to medication use. In a recent randomized clinical trial, the health behaviors of coronary patients did not improved markedly even after an education program compared with conventional care consisting only of a doctor’s prescription [38]. Based on our finding, strategies for the early detection of patients with low medication adherence and monitoring of medication use are important for the management of AMI because patients with poor medication adherence may have more difficulty with lifestyle modification.

Our finding also suggests that the healthy adherer effect should be considered in a clinical research aim to evaluate the effects of medications among patients with cardiovascular disease. Even in the RCTs of cardiovascular agent, findings of reduced mortality among good adherers of placebo are likely to be explained by healthy adherer effects [6]. Based on these results, if the experimental group has good adherence similar to that of the placebo group, the healthy adherer effect might result in over-estimated effects of preventive intervention on clinical outcomes even in clinical trial. Thus, researchers should consider the possibility of the healthy adherer effect and make efforts to reduce it when designing, analyzing, and interpreting the results in such studies on adherence and outcome.

It is noteworthy that, unlike MMS-knowledge, the MMS-motivation domain is associated with the adherence to lifestyle modification (Additional file 1: Table S1). Thus, to improve medication adherence and lifestyle modification, the specific methods for motivating patients to follow cardiac risk reduction recommendations seem to be important. It will be essential to expand communication between patients and their doctor [39] and use tailored education in order to promote the patient’s motivation in regards to their own medication. Recently, a considerable amount of attention has been focused on the application of new technologies such as computer-based education and mobile phone reminders to encourage patient responsibility for medications [40].

Among the demographic characteristics, only sex was associated with adherence to lifestyle modification in the multivariable analysis. In previous studies, factors such as sex, age, education, and income were known to be associated with health behaviors in patients with coronary artery disease. In the Reasons for Geographic and Racial Differences in Stroke study, being female and having a low educational level (<high school) and a low annual household income (< 20,000 dollars) were associated with low adherence to an ideal lifestyle after coronary artery disease [22]. In an Italian survey on Cardiac Rehabilitation and Secondary Prevention after Cardiac Revascularization, younger age was a predictor of smoking again after quitting, and older patients with comorbidity tended to persist in a sedentary lifestyle and poor diet after coronary revascularization [41]. In the Norwegian Coronary Prevention Study, current smoking was significantly more frequent in younger patients, and current smoking and low physical activity were significantly higher in females than in males after coronary artery disease [24].

Among the six CROQ subclasses in our study, lower symptoms and high satisfaction were associated with adherence to lifestyle modification among post-AMI patients. The finding is consistent with other previous studies. Greater medical burden, diabetes, depressive symptoms, low optimism/positive affect at baseline were associated with subsequent non-adherence to physical activity, healthy diet, and/or medications among patients with acute coronary syndrome [42]. The domains of quality of life associated with adherence to lifestyle modifications seem to differ according to the studies. In our study, CROQ-physical function was not significantly associated with adherence to lifestyle modification in both crude and multivariable-adjusted logistic models. However, in a previous study, the physical health domains in quality of life predicted adherence in cross-sectional and prospective analyses, whereas the mental health domain was not associated with adherence [20].

The strength of this study is that the results are generalizable compared with a single-center study because this study included the patients from 11 hospitals nationwide. In addition, we examined the healthy adherer effect in post-AMI patients with regard to HRQoL using the CROQ. The CROQ measures symptoms, physical functioning, psychosocial functioning, cognitive functioning, satisfaction, and adverse effects for patients who undergo coronary revascularization. This study has several limitations. First, information on the patients’ regular adherence to the six lifestyle modifications were obtained using a single self-report questionnaire with items corresponding to each of six lifestyle modifications behaviors; thus, the current study might be subjected to recall and measurement biases. However, in some ways, this simple questionnaire may be more applicable to the clinical setting. Second, participants had regular clinical visits at a particular university hospital; thus, it is possible they had a healthier lifestyle and improved adherence to their medication regimen. Therefore, patient lifestyle status may be overestimated compared with post-AMI patients in the general population. Additionally, there is a possibility of selection bias because patients with medical conditions who had difficulty in participating in the interview were excluded. However, we have tried to ensure that most of the patients who met the inclusion criteria participated in the study unless they were in very poor health or did not agree to participate. Third, a self-reported scale (i.e., MMS) was used in measuring medication adherence [43]; thus, we could not verify whether the participant took the prescribed drug or not. In future studies, the use of a more accurate medication assessment tool utilizing the detailed records of the hospital will be necessary. Additionally it is necessary to evaluate how the relationship among medication adherence, lifestyle modification, and HRQoL varies when considering the severity of AMI.

## Conclusions

In our study, medication adherence was statistically associated with lifestyle modification, suggesting a possible presence of a healthy adherer effect in post-AMI patients. The health adherer effect should be considered in observational studies as well as in clinical trials aimed at assessing the effects of medications among patients with a cardiovascular disease. Because medication adherence may be a surrogate marker for lifestyle modification among patients, physicians and caregivers should identify patients with poor medication adherence and monitor their medication use. Additionally, the implementation of motivational education programs is important to promote patient motivation regarding lifestyle modifications and medications.

## Additional file

Additional file 1: **Table S1.** Odds ratios on the adherence to health-related behaviors using logistic regression analysis. (DOCX 44 kb)

## Abbreviations

AMI: Acute myocardial infarction
CABG: Coronary artery bypass surgery
CROQ: Coronary Revascularization Outcome Questionnaire
HRQoL: Health-related quality of life
MACE: Major adverse cardiovascular events
MMS: Modified Morisky Scale
MOS: Medical Outcomes Study
PCI: Percutaneous coronary intervention
PTCA: Percutaneous transluminal coronary angioplasty
RCC: Regional Cardiocerebrovascular Centers
RCTs: Randomized clinical trials
STEMI: ST-Segment elevation myocardial infarction
