# Supplementary material for: Relationships among medication adherence, lifestyle modification, and health-related quality of life in patients with acute myocardial infarction: a cross-sectional study
Source: Health Qual Life Outcomes. 2018 May 22;16:100. doi: 10.1186/s12955-018-0921-z (PMC5964665; doi:10.1186/s12955-018-0921-z)
Supplement: Supplementary file 1 — Table S1. Odds ratios on the adherence to health-related behaviors using logistic regression analysis. (DOCX 44 kb) [file 12955_2018_921_MOESM1_ESM.docx]

Table S1. Odds ratios on the adherence to health-related behaviors using logistic regression analysis^a^ (n=417)

|  |  | unadjusted analysis | |  | adjusted analysis | |  | adjusted analysis | |  |
| --- | --- | --- | --- | --- | --- | --- | --- | --- | --- | --- |
|  |  |  | |  | model1 | |  | model2 | |  |
| characteristics |  | OR | 95% CI | p-value | OR | 95% CI | p-value | OR | 95% CI | p-value |
| sex | male (ref) | 1.00 |  |  | 1.00 |  |  | 1.00 |  |  |
|  | female | 2.40 | 1.42-4.06 | 0.001^*^ | 2.30 | 1.24-4.26 | 0.008^*^ | 2.37 | 1.23-4.57 | 0.010^*^ |
| age (years) | <50 (ref) | 1.00 |  |  | 1.00 |  |  | 1.00 |  |  |
|  | 50-59 | 1.98 | 0.97-4.03 | 0.969 | 1.61 | 0.77-3.37 | 0.856 | 1.86 | 0.87-3.99 | 0.721 |
|  | 60-69 | 2.52 | 1.26-5.04 | 0.162 | 1.81 | 0.84-3.91 | 0.408 | 1.98 | 0.89-4.41 | 0.481 |
|  | 70≤ | 3.14 | 1.57-6.30 | 0.007^*^ | 1.99 | 0.87-4.57 | 0.243 | 2.43 | 1.01-5.80 | 0.140 |
| marital status | married | 1.46 | 0.92-2.34 | 0.112 | 1.89 | 1.09-3.26 | 0.023^*^ | 1.68 | 0.95-2.96 | 0.073 |
|  | single/others ^b^ (ref) | 1.00 |  |  | 1.00 |  |  | 1.00 |  |  |
| education | ≤ middle | 1.50 | 1.00-2.24 | 0.050 | 1.09 | 0.64-1.84 | 0.757 | 1.19 | 0.69-2.05 | 0.541 |
|  | ≥ high (ref) | 1.00 |  |  | 1.00 |  |  | 1.00 |  |  |
| family income | ≤100 (ref) | 1.00 |  |  | 1.00 |  |  | 1.00 |  |  |
| (10,000 won/month) | 101-200 | 1.05 | 0.57-1.90 | 0.146 | 1.10 | 0.57-2.15 | 0.308 | 0.99 | 0.48-2.02 | 0.475 |
|  | 201-300 | 0.61 | 0.34-1.07 | 0.159 | 0.72 | 0.36-1.43 | 0.303 | 0.64 | 0.31-1.32 | 0.196 |
|  | ≥301 | 0.60 | 0.36-1.00 | 0.096 | 0.77 | 0.39-1.53 | 0.490 | 0.78 | 0.38-1.61 | 0.739 |
| MMS-knowledge | 0-1(ref) | 1.00 |  |  | 1.00 |  |  | 1.00 |  |  |
|  | 2 | 3.66 | 1.02-13.1 | 0.029^*^ | 2.20 | 0.53-9.08 | 0.245 | 2.00 | 0.44-9.14 | 0.277 |
|  | 3 | 2.84 | 0.79-10.21 | 0.270 | 1.94 | 0.47-7.97 | 0.495 | 1.60 | 0.35-7.31 | 0.772 |
| MMS-motivation | 0-1(ref) | 1.00 |  |  | 1.00 |  |  | 1.00 |  |  |
|  | 2 | 1.92 | 0.84-4.36 | 0.853 | 1.92 | 0.79-4.64 | 0.685 | 2.14 | 0.86-5.33 | 0.508 |
|  | 3 | 3.30 | 1.59-6.88 | <0.001^*^ | 2.85 | 1.28-6.35 | 0.006^*^ | 2.98 | 1.31-6.82 | 0.008^*^ |
| CROQ-symptoms | | 1.03 | 1.02-1.05 | <0.001^*^ |  |  |  | 1.02 | 1.00-1.04 | 0.017^*^ |
| CROQ-physical functioning | | 1.01 | 0.99-1.02 | 0.309 |  |  |  | 1.00 | 0.99-1.02 | 0.879 |
| CROQ-psychosocial functioning | | 1.01 | 1.00-1.03 | 0.009^*^ |  |  |  | 1.00 | 0.98-1.01 | 0.550 |
| CROQ-cognitive functioning | | 1.01 | 1.00-1.03 | 0.046^*^ |  |  |  | 1.01 | 0.99-1.02 | 0.484 |
| CROQ-satisfaction | | 1.02 | 1.01-1.04 | <0.001^*^ |  |  |  | 1.02 | 1.01-1.04 | 0.009^*^ |
| CROQ-adverse effects | | 1.02 | 1.00-1.04 | 0.017^*^ |  |  |  | 1.02 | 1.00-1.04 | 0.083 |

^a^binary outcome variable was higher adherence to lifestyle modifications, that was defined as a subject who are adherent to five or more among the six health behaviors including low-salt intake, low-fat diet and/or weight-loss diet, regular exercise, stress reduction in daily life, drinking in moderation, and smoking cessation; ^b^ others included separated, divorced, and widowed status. ^*^*p*-value <0.05
